# A Phenomenological Study of Older Individuals' Experiences of Safety at Home

**DOI:** 10.1111/scs.70165

**Published:** 2025-12-09

**Authors:** Kivimäki Taina, Stolt Minna, Charalambous Andreas, Katajisto Jouko, Suhonen Riitta

**Affiliations:** ^1^ Department of Nursing Science University of Turku Turku Finland; ^2^ Wellbeing Services County of Satakunta Pori Finland; ^3^ Department of Nursing Cyprus University of Technology Limassol Cyprus; ^4^ Department of Mathematics and Statistics University of Turku Turku Finland; ^5^ Turku University Hospital Wellbeing Services County of Southwest Finland Turku Finland

**Keywords:** experiences, home, interview, older individuals, phenomenological, safety

## Abstract

**Aims and Objectives:**

To identify the elements of safety for older individuals living at home and how they describe safety as a lived experience.

**Methodological Design and Justification:**

The research design was based on a phenomenological inquiry. The narratives were older individuals' own experiences of safety at home.

**Ethical Issues and Approval:**

The study followed Finnish law and the European Code of Conduct for Research Integrity. The university committee of ethics gave ethical approval, and permission to conduct the study was granted from one wellbeing services county.

**Research Methods:**

Ricœur's Hermeneutic Phenomenological Analysis method was used to examine 16 older individuals' life experience of safety at home. The data was collected using a semi‐structured interview framework and two structured instruments.

**Results:**

Safety at home was expressed in many ways as being safe, living safely, and having a safe feeling with other people and most often in a positive manner. Older individuals' narratives of safety related to safe living in one's own home; being able to take care of oneself; reminiscing and longing; living at home is meaningful and valuable; changes in physical functioning; getting help from homecare and others.

**Study Limitations:**

The selection of participants was approached by homecare professionals and contact persons, who recruited older individuals to participate in the study. Whilst the inclusion criteria were known, the selection time was short, so not all potential participants were reached within this time.

**Conclusions:**

For older individuals, safety at home means prerequisites for their daily life, including their unique perspectives and lived experiences. There is a need to explore diverse cultural contexts and employ longitudinal approaches to investigate how factors such as closeness to nature or social isolation contribute to older individuals' experiences of safety in their living environment.

## 1 Introduction

Safety at home is a multifaceted phenomenon that can affect the ability of older individuals to cope at home [1, 2, 3, 4]. As a complex phenomenon, safety includes physical, social, emotional and mental dimensions as well as the element of cognitive safety [4]. For older individuals, living at home involves physical, psychosocial, environmental and socio‐economic prerequisites [5]. Practically, safety at home for older individuals includes the consideration of issues such as medication management [2, 4], the prevention of falls [4], home renovation [4], the use of technology [6], relationships with other people [7], feeling secure at home [4], and a decline in cognitive functioning [4]. When the physical or mental capacity of an older individual is impaired and threatens their safety and functioning in everyday life, the individual's living conditions at home should be evaluated and adapted [4, 5].

People worldwide are living longer. Between 2015 and 2050, the population of over‐60‐year‐olds is expected to double from 12% to 22%, and the amount of older people will double (2.1 billion). The number of persons aged 80 years or older is expected to triple between 2020 and 2050 to reach 426 million [8]. For example, action plans for housing for older individuals continue to develop, with solutions including the construction of community homes together with municipalities in Finland. The use of technology, artificial intelligence and robotics to support the wellbeing, health promotion, and care of older individuals will increase in the future. Smart technologies that support independent performance can be used to improve the everyday lives of older individuals, for example, in long‐term professional homecare services and in a variety of preventive activities [9]. These technologies can benefit older individuals, who prefer to live in their own homes rather than move into a senior home or 24‐h service housing [10].

### 1.1 Safety at Home

Regarding physical safety at home, accidents due to falling, slipping, or fire have been found to be the main worries for older individuals, and higher age has been linked to experiences of falls and slips [11]. Therefore, preventive measures including training programs have been practiced by older individuals at home [11]. When faced with the use of gerontechnology (e.g., safety bracelets, smartphones, health monitoring devices, automated medicine dispensing), older individuals have stated that financial resources, a lack of support and ignorance in the operation of the devices have limited their approval of the devices [6, 12].

Sometimes long‐term health problems, including symptoms of memory disorders, create difficulties for older individuals in viewing long‐term professional homecare services as a part of safety at home as well as self‐experienced health care [4, 11]. Additionally, accurate medication therapy taking has an impact on older individuals' long‐term health [2]. Older individuals tend to see their home as a place where they are safe and free to decide who can be welcomed in [13]. In one study based on interviews, older individuals expressed that long‐term professional homecare staff had been friendly and helpful, that help had been given when needed, and that these services had an impact on their safety at home. The older individuals felt that the quality of services and the feeling of safety were related [11].

Older individuals may experience loneliness towards the ends of their lives when social relationships decline [7, 14]. It is reported by WHO [15] 20%–34% of older individuals, for example in China, Europe, Latin America and the United States of America, are lonely. Social isolation and loneliness are harmful. They shorten older individuals' lives and damage their mental and physical health and quality of life [15].

Older individuals tend to frame their loneliness only in negative terms [16]. When revealing their vulnerable thoughts, they have expressed feelings of helplessness, worthlessness and existential loneliness, and have conveyed the idea that their existence is disconnected from the world [17]. On the other hand, older individuals experience their home positively, as a place full of memories [10], and nostalgic memories can support feeling safe in healthy aging [18]. Older individuals often see living alone as being an inevitable consequence of living a long life [5]. Access to nature has been found to be important to older individuals, as it provides both physical and emotional benefits for them [19].

Older individuals' needs and expectations are known in safety issues, especially concerning home renovations increasing accessibility [20], fire safety [4], medication management [2, 4], care services [3, 4] and gerontechnology [6, 12]. However, there is a significant gap in research about older individuals' experiences of multidimensional safety or feelings of safety at home. Safety at home in this context is rarely studied based on lived experience but instead on separate safety incidents. In the speech of professionals, research evidence about safety is related to the physical dimension, but older individuals describe the sense of safety more broadly. It is important to identify the factors linked to their subjective experiences. This could be used to promote, understand and interpret the meaning of safety at home for older individuals, which could be described as existential safety.

The aim of this study was to identify the elements of safety for older individuals living at home and how they describe safety as a lived experience.

Research questions:

1. What are the elements of safety at home as expressed by older individuals?
2. How do older individuals describe safety at home as a lived experience?

## 2 Methods

This study was part of a larger mixed‐methods study investigating the experience of safety of older individuals at home. The research design was based on phenomenological inquiry. The study was conducted and reported in line with the Consolidated Criteria for Reporting Qualitative Research (COREQ) [21].

The data were collected from older individuals at home using an interview framework and two structured instruments. The narratives included older individuals' own experiences of safety at home. The interviews were recorded and transcribed verbatim.

### 2.1 Data Collection/Interviews

Individual interviews were conducted in April and May 2023 in western Finland. The interviews consisted of structured background questions and open‐ended questions about safety at home. Results related to the health status and quality of life of the older individuals are represented in numbers (Table 1).

**TABLE 1:** Interview framework about safety at home.

Interview framework	
Sociodemographic data	
Age	
Gender	
Home care services	
Marital status	
Place of residence	
Housing conditions	
Dwelling place	
Form of ownership	
Descriptions of home (e.g., accessibility)	
EQ‐5D‐3L/structured	
Perceived health state (health status of the older individual on the day of the interview)	
WHOQOL‐BREFF/structured	
Quality of life (older individuals assess their quality of life over the 2 weeks preceding the interview)	
Open‐ended questions	
What living at home and safety means to you as an older individual?	
2 What is the importance of relatives, and friends for safety at home and taking care of finances, hobbies?	
3 Do you feel safe at home?	
4 Do you accept help from relatives or others at home?	
5 Do you take care of yourself?	
6 What else influences safety at home?	
7 What helps safety at home?	
8 What does not help safety at home?	

Two structured instruments were used in the interview, EQ‐5D‐3L and WHOQOL‐BREF. The index values derived from these instruments were used as background information for the participants. The EQ‐5D‐3L questionnaire, developed by the EuroQoL Group, is a standardised measure of perceived state of health [22]. It consists of a classification system of five dimensions (mobility, self‐care, usual activities, pain or discomfort, and anxiety or depression) that are subjectively rated with a visual analogue scale from 0 (worst imaginable health) to 100 (best imaginable health) (EQ VAS). In the EQ‐5D‐3L, each dimension is described with three severity levels (1 = no problems; 2 = some problems; 3 = extreme problems). The instrument provides a variety of information in terms of a descriptive health profile, an individual EQ VAS rating, and an index score [23].

The WHOQOL‐BREF, a brief generic questionnaire to assess quality of life [24], includes four dimensions (physical, mental, social and environment) for which scores are calculated on a scale from 0 to 100. In addition, the instrument contains two questions, scored on a scale of 1 to 5, concerning overall quality of life and health status. A higher score means better quality of life, both for individual issues and for different dimensions [25]. The data were analysed using SPSS 26.0 (IBM Corporation).

The first researcher with previous experience in conducting interviews with long‐term professional homecare clients interviewed older individuals individually or in the presence of an informal caretaker. Participants did not know the researcher before. The interviews took place in the homes of the older individuals, where they described their experiences of safety at home through narratives. Everyone was interviewed once, and the interview sessions lasted between 33 and 120 min.

### 2.2 Eligibility and Recruitment

Participants were eligible to participate if they were (a) aged 65 years or older, (b) receiving regular public long‐term professional homecare services, and (c) living independently at home or in communal housing. Participants receiving home‐based support services, such as meals, transport, or washing/hygiene services were excluded.

The wellbeing services county consists of several home care areas. Each home care area named a contact person who worked in close collaboration with the first researcher. The nine contact persons recruited the potential participants according to the study guideline and inclusion criteria. These contact persons asked several older individuals receiving regular professional homecare about their willingness to participate in the study. Those willing to participate in the interview gave their oral consent to the contact persons, allowing the researcher to contact them by phone to schedule an interview. Prior to the interview at home, the older individual was again informed about the study and its significance, and written informed consent was obtained to participate in the study.

### 2.3 Participants

Nineteen older individuals gave preliminary oral consent, of whom one individual died just before the interview and two refused to participate. Therefore, a total of 16 older individuals were interviewed: 13 women and 3 men in long‐term professional homecare. Their ages varied from 72 to 95 years (mean = 85.6 years), and they lived in urban or rural areas. Their marital status included being a widow (*n* = 9), married (*n* = 4), single (*n* = 2), or divorced (*n* = 1). Fourteen participants lived alone, and two lived with a spouse (informal caretaker). Nine participants lived in rented apartments, one had their own apartment, and five participants had their own houses. Using the EQ‐5D‐3L, the older individuals' assessments of their own perceived state of health on the day of the interview were between 0.09 and 0.86, which indicated a range from problems in each dimension to almost full health. In addition, their health evaluations ranged from 35 to 90, from poor to exceptionally good, when measured with the subjectively rated visual analogue scale. The participants rated their quality of life in the previous 2 weeks (WHO‐QOL‐BREF) from 55.4 to 81.77, from fair to good (Table 2).

**TABLE 2:** Sociodemographic information of participants in long‐term professional homecare.

Participants	Age	Gender	Marital status	Place of residence	Housing conditions	Dwelling place	Form of ownership	EQ‐5D‐5L (reference value)	VAS‐scale	WHOQOL‐BREF
Edna	83	Female	Widow	Rural area	Single	Detached house	Own occupied house	0.86	80	71.09
Steve	76	Male	Married	Rural area	With spouse	Detached house	Own occupied house	0.09	50	67.71
Helen	93	Female	Widow	Rural area	Single	Terraced houses	Rented apartments	0.39	65	62.24
Ronald	75	Male	Single	Rural area	Single	Terraced houses	Own occupied flat	0.45	60	55.47
Imogen	88	Female	Widow	Town	Single	Detached house	Own occupied house	0.74	90	75.00
Evelyn	83	Female	Divorced	Town	Single	Apartment house	Rented apartments	0.54	55	78.65
Rose	88	Female	Married	Town	Single	Apartment house	Rented apartments	0.63	40	65.63
Violet	93	Female	Widow	Town	Single	Apartment house	Rented apartments	0.45	50	63.54
Alice	72	Female	Widow	Town	Single	Apartment house	Rented apartments	0.45	70	76.82
Lorraine	89	Female	Widow	Rural area	Single	Apartment house	Rented apartments	0.83	85	71.09
Robert	90	Male	Married	Rural area	Single	Apartment house	Rented apartments	0.45	85	67.71
Lily	95	Female	Single	Rural area	Single	Apartment house	Own occupied flat	0.45	75	57.81
Peggy	77	Female	Widow	Town	Single	Apartment house	Rented apartments	0.75	80	75.52
Kathleen	85	Female	Widow	Town	Single	Terraced houses	Rented apartments	0.46	35	81.77
April	94	Female	Widow	Town	Single	Detached house	Own occupied house	0.63	80	70.57
Lydia	89	Female	Married	Town	With spouse	Detached house	Own occupied house	0.69	85	73.18

#### 2.3.1 The Study Environment: Long‐Term Professional Homecare and Services

In 2022, around 59% (114,000 customers) of all long‐term professional homecare customers received regular long‐term professional homecare services in Finland. Of these, most of the regular long‐term professional homecare services were provided to older individuals aged 85 to 94 years old (39%) [25]. About half of regular long‐term professional homecare customers received at least one home visit per day. In 2020, 50% of those who used a lot of long‐term professional homecare services had a full‐year homecare period [26]. When an older individual needs care in Finland, it is possible to get it at home or in a home‐like environment. Often, long‐term professional homecare and other support provided at home consist of services provided by public and private parties and organisations. A network consisting of professionals, family members and volunteers can make it possible for an older individual to live in their own home [27].

### 2.4 Interpretation

Ricœur's [28] hermeneutic phenomenological analysis method was followed including naïve reading, structural analysis and comprehensive understanding. Listening to older individuals and their narratives about safety at home was the starting point to understanding the dialogue and transferring their experiences of safety at home to the researcher [28, 29, 30]. The most important thing is to form an understanding of an individual's lived experience and understanding it [28, 29, 30, 31]. Older individuals described personally significant multidimensional aspects of safety at home. One individual had thought about safety issues in advance; the other told the things that had been bothering her for a long time in life. The importance of words and understanding safety was emphasised when interpreting safety in the home of an older individual.

Naïve reading was used during transcription to gain an understanding of the meanings of safety at home. A structural analysis was conducted with a qualitative research data analysis tool (NVivo software) for organising and managing data. The interviews were transferred in full to NVivo. Using the NVivo codes based on meaning units of words and sentences related to safety at home, similar aspects of safety at home were combined into sub‐themes and eventually main themes (Table 3). Finally, a comprehensive understanding of older individuals' experiences of safety at home was created when interpreted with existing knowledge.

**TABLE 3:** Sub‐themes and themes of safety at home experienced by older individuals.

Sub‐themes	Themes	Main theme
Aging at one's own home Being close to nature The best neighbourhood Anxiety as a human emotion Feeling of safety Home renovation Fire safety Gerontechnology	Safe living in one's own home	Safety at home experienced by older individuals
Participation in decision‐making	Being able to take care of oneself	
Reminiscing and longing	Reminiscing and longing	
Life is meaningful and valuable	Living at home is meaningful and valuable	
Movement or ability to move Ailments affecting the ability to move	Changes in physical functioning	
Long‐term professional home care Next of kin Neighbours	Getting help from homecare and others	

### 2.5 Ethical Considerations

Older individuals were fully informed about the study both orally and in writing. This information included background information about the study, the voluntary nature of participation, information about the research provider, the purpose of the study, implementation of the study, potential benefits of the study for the participants, disadvantages and inconveniences that may arise from the study, confidentiality and data protection, and research costs and financial reports. This information was given, first, when the contact person contacted the potential participants and, second, at the beginning of the interview at home by the researcher. All participants gave their signed informed consent. It was possible for them to cancel the interview or withdraw from the study if they so wished. The study followed the Finnish Law [32] and the European Code of Conduct for Research Integrity [33]. The university committee of ethics (35/2022) gave ethical approval, and permission to conduct the study was granted from one wellbeing services county (SHVA/510/1301/2023).

## 3 Findings

### 3.1 Naïve Reading

Older individuals revealed experiences of safety at home in their narratives. Safety at home was expressed in many ways as being safe and living safely, having a safe feeling with other people and most often in a positive manner. Everyday life went well with the help of homecare services, relatives and friends in general. Everyone wanted to stay at home as long as possible; living elsewhere was not seen as a good option. Their narratives of safety at home were related to living at home, the home environment, coping at home, experiencing illness and mood. The older individuals thought they were nearing the end of their lives and had accepted their life situation. They missed their deceased children, spouses and friends. Some older individuals reflected on the significance of their own lives, some were lonely and some longed for someone to talk to.

### 3.2 Structural Analysis

Based on the structural analysis, six main themes and 16 sub‐themes emerged. Themes identified derived from the data. These were assembled into six themes: safe living in one's own home; being able to take care of oneself; reminiscing and longing; living at home is meaningful and valuable; changes in physical functioning; getting help from homecare and others (Table 3).

### 3.3 Theme 1. Safe Living in One's Own Home

According to the participants, safe living at home was associated with aging at one's own home, the importance of being close to nature, living in the best neighbourhood, anxiety as a human emotion, the feeling of safety, home renovation, fire safety and gerontechnology (Table 4).

**TABLE 4:** Example of the interpretation of theme ‘Safe living one's own home’.

Meaning unit	Condensation	Sub‐theme	Theme
Home: ‘there's nothing to complain about’ (Lily)	Home is a safe, comfortable place	Aging at one's own home	Safe living one's own home
Fire safety: ‘has just been completed and is up to date’ (Robert)	Fire safety	Fire safety and gerontechnology	
Nature: ‘we have a rocky hill. I can move when I know those rocks. I do not move except on a level yard these days’ (April)	The importance of nature, yard and animals	The importance of surrounding nature	
Neighbours: ‘on the first day after the move a neighbour came with a cake’ (Kathleen)	Helpful, kind and friendly neighbours	The best neighbourhood	
Anxiety: ‘There will be anxiety when there are no friends, and you will feel bad. It's a panic’ (Rose)	Experiencing anxiety	Anxiety is a human emotion	
Feeling of safety: ‘I have one upstairs, it's so safe. Trust that safety, yeah. I'll have peace there. I tell you, when you're safe in God, you're better off. I have that kind of safety that I trust in if anything happens to me. I won't worry about it. It is important when you are allowed to be like yourself, not to ask anyone’ (Kathleen)	Feeling of safety or unsafety	Feeling of safety	
Home renovations: ‘I have lived here for 16 years; there will be a lot of renovations. It has been said many times that the balcony will be renewed. Just done plumbing renovation, cost a lot’ (Peggy)	The need for renovation at home	Home renovations	

Aging at one's own home referred to the individual's home being seen as a safe and comfortable place to live. One participant had been discharged from the hospital and moved directly to communal living, as this was their best option. Some of the participants thought that service housing with 24‐h assistance was not a good place for older individuals. They felt that there was nothing to complain about in their current home, and they wanted to live there as long as possible.

When you think about age, it doesn't get any better than this place, in this context. (Rose)

Being close to nature felt significant for older individuals. Having a window view of nature was important. Watching birds and other animals was a pleasant activity for the older individuals and a kind of daily routine. One participant, who could walk, said there was a 1‐km‐long forest trail near their home. They could experience birds and nature, which was enjoyable. This older individual was worried about the maintenance of the trail: in the winter it had been icy, and they were wondering about the current situation. The participants also shared concerns about the future, about whether human beings would ruin their own environment.

I enjoy sitting outside and looking at the squirrels and the birds wandering around. (Imogen)

Older individuals felt they had the best neighbourhood. Some neighbours had come to greet them, bringing a cake, when the older individual had moved into the new home. The participants described neighbours as usually being helpful, kind and friendly towards older individuals. A neighbour might have a universal key to an older individual's apartment. Participants felt that they could call a neighbour anytime. The older individuals tended to have good relationships with all the neighbours, when possible.

I have the best neighbour, moved here a couple of years ago. A great, nice person. (Lily)

Anxiety as a human emotion was expressed sometimes as the older individuals worried about things such as their children's wellbeing. They described feeling bad or panicked and that they could not be themselves. Although having a worse day they did not get anxious. This feeling was sometimes prevented by taking a painkiller.

I have agony in my chest, I'm afraid that I'll feel the anxiety and then the depression. (Edna)

There was a positive feeling of safety at the current stage of life at home. Some had faith in God and God's protection while some feared strangers. Some had a hammer hid near the window for protection. However, they generally felt that living at home was safe. The feeling of safety was so strong that some even left the door unlocked so that they would not have to get up to open it for visitors.

Home renovation was usually not needed, but some older individuals needed surface refurbishment. For older individuals, the renovation of the balcony to reach there even in winter seemed to take ages. Another concern raised was high thresholds in a hallway and bathroom in one home. A threshold of about 7 cm in height prevented one older individual who always used a wheelchair from safely entering and exiting the bathroom. This individual therefore tried to restrict her visits there to 3–4 times a day.

Fire safety was related to fire alarm systems, fire blankets and sprinklers on the ceiling. Most of the older individuals stated that their fire safety system had been recently checked and was working properly. In some homes, fire alarm systems were inactive due to dead batteries or were simply broken. If the older individual lived in an apartment building or communal housing, fire safety was taken care of by someone else. Gerontechnology included safety bracelets, smart locks, smart phones and telecare. One of the older individuals was expecting to get an electronic pill dispenser from their long‐term professional homecare service.

### 3.4 Theme 2. Being Able to Take Care of Oneself

Older individuals participated in the decision‐making about their affairs together with their relatives and homecare professionals. According to the older individual living in communal housing, resident meetings allow them to express their opinions. On the other hand, one older individual said that she had been informed about a service decision that had not been discussed in a previous meeting. In some cases, the children of the older individuals made decisions.

Together with the children, we discuss and then discuss payments with homecare. (Peggy)

The older individuals had not always told their family members about their difficulties in taking care of themselves at home. They had tried to cope as well as possible and independently at home.

It's not always that, but I try to pretend to myself and people that, to myself most of all, that I can do it even though I can't do it anymore. (Edna)

Family members often managed the older individuals' financial affair. The participants were concerned about the cost of living.

### 3.5 Theme 3. Reminiscing and Longing

For older individuals, reminiscing was considered natural at this stage, as was longing. They could have gotten support in the aging process from friends if they had been alive. It was mood‐uplifting to remember the good old days fondly and long for important conversations. They felt closer to their deceased friends or family members. This increased their feeling of safety at home. Furthermore, some had friends living in another locality, and it had become challenging to visit them as their strength dwindled. They stayed connected with telephone.

The older individuals had been active when they were younger. They told stories of participating in sports, acting, singing in a choir and writing plays, among other things. They expressed feeling tired but still longing for these active times. They remained interested in reading. One was writing a third book about their childhood memories, and another was interested in gardening.

### 3.6 Theme 4. Living at Home Is Meaningful and Valuable

The older individuals felt their life was meaningful and valuable as they were enjoying life and were satisfied with themselves at home. They found meaning in their children and grandchildren and in generally having a good life. One older individual had been appreciated as a playwright. Another said relatives had been interested in her, a single person. It was important for one older individual to get up in the morning at their own pace and enjoy themselves. One expressed being satisfied with life and what had been given to her and accepted it with gratitude. On the other hand, some implied that their advanced age was an obstacle to knee operation, for example.

When you know you won't be walking around much longer, and when no one expects me to do this anymore, when I can't help anyone anymore. (Violet)

### 3.7 Theme 5. Changes in Physical Functioning

Changes in physical functioning included movement or ability to move and ailments affecting the ability to move. Older individuals were no longer able to move actively and independently in the same way as before. The weakening of movement had led to falls, for example, on the outdoor stairs, in the toilet, and even with a rollator. As a result of a fall, one older individual had broken a hip. To prevent such falls, some older individuals did walk exercises, and all of them had some kind of aid for secure movement.

The older individuals told about their ailments affecting their ability to move, such as multiple sclerosis (MS), Arteriosclerosis Obliterans (ASO), heart disease, stroke, suffering from polio as a child, severe kidney disease, macular degeneration, back surgery, diabetes, asthma, hypertension, Parkinson's disease and, in the case of one older individual, suffering twice from COVID‐19. The older individuals also reported pains in the hands, feet, knees, and back and during wound care. The pain was described as constant, feeling electric or post‐convulsive after wound treatment. With weakened muscles, one older individual could use only one hand to help the other. Older individuals had adapted to their existing ailments, but some of them no longer bothered to go to the doctor, with appointments seeming pointless.

Twice affected by corona and after that a heart attack, the hand does not work, cannot get my hands up to, for example, hang laundry to dry. (Peggy)

### 3.8 Theme 6. Getting Help From Homecare and Others

Older individuals were receiving help from long‐term professional homecare, next of kin, or neighbours. Usually, it was easy to ask for help from next of kin. It was sometimes hard to ask homecare staff to help more as functioning weakened.

I don't have a problem as I have a good relationship with my daughter, but if you have to ask a stranger, it might prevent something from progressing. (Evelyn)

Homecare staff was described as nice, helpful and dependable. In many cases they took care of medication management. The older individuals wished that homecare staff would sometimes spend a longer time visiting, but on the other hand they understood they were busy.

## 4 Discussion

### 4.1 Comprehensive Understanding

This study aimed to identify the elements of safety for older individuals living at home and how they describe safety as a lived experience. To achieve these goals, a comprehensive understanding was achieved by moving back and forth between explanation and understanding, following a hermeneutic arc and taking multiple sources of knowledge into consideration. The comprehensive understanding can be used to inspire new possibilities for the care of older individuals [28, 29, 30, 31].

Safety at home was described through six main themes: safe living at one's own home; being able to take care of oneself, reminiscing and longing; living at home is meaningful and valuable; changes in physical functioning; getting help from homecare and others. In this study, the older individuals themselves were allowed to share their feelings, experiences, and thoughts about their future related to their lives and safety at home. The narratives drew a picture of the older individuals' lives and coping at home. For professionals, this means new ways to approach safety at home and promote a holistic understanding of safety instead of focusing on physical safety [1, 2, 4, 20].

Older individuals strongly expressed the importance of staying connected and having relationships with others [4, 5, 11, 13]. One should be concerned about those who do not have close relatives, friends, or neighbours to care for the feeling of safety at home. It is important to identify their emotional and mental needs concerning living at home. In our study, one older individual underlined that 24‐h assistance did not feel like home, and he could never call it home. In this case, the home environment was unsatisfactory even though the living itself was safe. This could also be influenced by the individual's separation from his spouse or whether he was ashamed of how the disease had changed him [16]. He had to move away from his home and live alone in this environment. He did not feel he would belong to the family anymore. This older individual seemed to lack the emotional and social safety that is important for everyone.

Older individuals expressed their preferences for living at home as more physical than emotional factors [15], for example, lifestyle and environmental elements. Older individuals often associate safety at home with physical aspects such as safety equipment, gerontechnology and access to homecare services. The home was seen as a protection for the older individuals and their possessions. They had refurbished their homes so they could cope with living there [2, 6, 11, 12].

Changes in physical functioning and existing ailments being knowledgeable vulnerabilities impacted the ability to move [11]. Impairment of mobility was associated with falls and injuries. These factors had contributed to older individuals ending up in homecare services. Almost all the older individuals had decreased vision, and almost all wore glasses. A few had impaired or completely gone vision due to eye disease and needed help with daily activities at home. However, these older individuals wanted to live in a familiar home environment and were ready to accept more services. In general, the home care staff were considered comfortable, helpful and reliable, but the length of their visits was considered too short.

Whether an older individual lived in a detached house, apartment building, or communal housing, the importance of nature was great. More forcefully than before, the connection to the place of residence and the community environment was highlighted, which was more significant than has been previously identified [19]. Being a part of familiar nature and community added meaning to the days and created a sense of safety. Furthermore, older individuals were worried about the global changes affecting nature and the climate now and in the future. Regarding the home environment, they mentioned worries about neighbours and that a good neighbour is important to being safe at home.

Some older individuals felt sad and anxious, not typical for them. They attributed the feeling of anxiousness to worries about their children, for example, congruent to Larsson [11]. Emotions such as these can hinder safety [4]. For many older individuals, the world outside the home may seem challenging or scary at times, but in this study, older individuals did not experience this as a threat to safety.

Housing was secured by various means, such as neighbouring assistance. Some expressed trusting in God for safety, something creating positive emotions for older individuals and promoting safety at home. Some, but not all, older individuals felt their lives were meaningful and valuable. For some, existential loneliness, sometimes resulting from losing close relationships, can result in life feeling like it has no meaning because they feel they do not matter to anyone else anymore [16]. These kinds of feelings seem to impact safety at home negatively. On the other hand, the older individuals were not ashamed, for example, of their physical aging, as Larsson et al. [16] noted. In this study, most of them had accepted aging as part of the course of life.

Participation in decision‐making is a positive outcome of safety at home, but decision‐making is not always done with older individuals. It is understandable that decreased health status prevents participation in decision‐making [4]. The interviewed older individuals did participate in decision‐making with their relatives and homecare professionals. The discussions gave the impression that in a rapidly changing world, such as that of technology, it was difficult to decide about something you do not know. As a result, they were trying to cope as well as possible and independently at home and considered it highly important that they will be consulted.

Reminiscing and longing are common among older individuals and can have both positive and negative effects on emotional safety. The older individuals longed to have conversations with former friends. This feeling sometimes leads to existential loneliness, as was expressed in this study. Loneliness as an emotional feeling has been found to have profound consequences for the physical and mental health of older individuals [15], so it also has an impact on the safety of older individuals. In this study, the older individuals may have chosen to live alone but not live in complete social isolation. Many older individuals mentioned loneliness being occasionally present and others being always present. They had experienced existential loneliness, but they were not suffering from it, unlike in a previous study [16]. Living alone or being alone sometimes fostered the feeling of lacking meaning, purpose, or value in life. It should be noted that the older individuals were generally satisfied, and these feelings were only one part of their lives related to emotional safety. The older individuals had resigned themselves to their fate of having lost close relationships.

According to the findings, the older individuals considered living at home or aging in place to be the most important element and best solution for their lives as they aged, exemplifying the importance of evaluating the safety environment of the home, holistically and existentially, with the older individual.

### 4.2 Implications

The findings of the study suggest implications for social‐ and healthcare providers to consider the experience of older individuals as the specialists of safety at home. Policy makers could use this knowledge to develop effective programmes for safety at home as older individuals wish to live in their homes longer in the future. Educators could create effective learning for students in caring and nursing studies taking into account, for example, the relation of emotional and mental safety and ageing.

### 4.3 Limitations

The selection of participants was approached by homecare professionals and contact persons who recruited older individuals to participate in the study. Whilst the inclusion criteria were known, the selection time was short, so not all potential participants were reached within this time. The data was rich—structural analysis provided meaningful content and structure and linked with existing knowledge built on that.

In the case of a large area of research, care must be taken to ensure the length of the selection period for those participating in the research. This highlights personal, subjective knowledge from an older individual's perspective. Lived experience of safety at home cannot be generalised.

### 4.4 Conclusions

For older individuals, safety at home means prerequisites for their daily life, including their unique perspectives and lived experiences. Future research should examine how loneliness affects emotional and mental safety, and how it influences the overall sense of safety at home. There is a need to explore diverse cultural contexts and employ longitudinal approaches to investigate how factors such as closeness to nature or social isolation contribute to older individuals' experiences of safety in their living environment. More ways for identifying the feeling of safety and building tools for identifying when comprehensive need assessment would be done for aged individuals living at home are needed.

## Author Contributions

K.T. conceived the study and collected data. K.T., S.M., C.A., and S.R. wrote the manuscript. K.T. and K.J. performed the statistical analysis. K.T., S.M., C.A., and S.R. edited the text. All authors approved the final submission.

## Funding

This study was supported by The Finnish Nursing Education Foundation (SHKS) and Konung Gustaf V:s och Drottning Victorias Stiftelse.

## Conflicts of Interest

The authors declare no conflicts of interest.

## Data Availability

Research data is not shared.
